# Study on the Isotherms, Kinetics, and Thermodynamics of Adsorption of Crystal Violet Dye Using Ag-NPs-Loaded Cellulose Derived from Peanut-Husk Agro-Waste

**DOI:** 10.3390/polym15224394

**Published:** 2023-11-13

**Authors:** Ghalia Saleem Aljeddani, Reem Mohammad Alghanmi, Ragaa A. Hamouda

**Affiliations:** 1 Department of Biology, College of Science, University of Jeddah, Jeddah 21589, Saudi Arabia; gsalgdanee@uj.edu.sa; 2 Department of Chemistry, College of Science, University of Jeddah, Jeddah 21589, Saudi Arabia; rmalghanmi@uj.edu.sa; 3 Biology Department, College of Science and Arts at Khulis, University of Jeddah, Jeddah 21959, Saudi Arabia; 4 Microbial Biotechnology Department, Genetic Engineering and Biotechnology Research Institute (GEBRI), University of Sadat City, Sadat City 32897, Egypt

**Keywords:** crystal violet (CV), *Azadirachta indica*, peanut husks, agro-waste, nanocomposites

## Abstract

A huge amount of textile dyes are released as industrial waste into the environment each year, which alters the water’s natural appearance and causes toxicity and carcinogenicity in the human body. Peanut husk is considered an agro-waste and contains many valuable compounds, such as cellulose. Different concentrations of cellulose were extracted from peanut husk and then loaded with bio-silver nanoparticles, which were fabricated using neem leaves (*Azadirachta indica*) as a reducing agent to form Ag-cellulose nanocomposites (Ag-Cell-NCMs). Different devices were used to characterize Ag-Cell-NCMs. The TEM images displayed that the size of Ag-Cell-NCMs ranged between 13.4 and 17.4 nm after dye adsorption. The Ag-Cell-NCMs were used to adsorb toxic dyes such as crystal violet (CV). Different parameters were applied, such as the ratio of cellulose to Ag-NPs, pH, contact time, adsorbent dose, dye concentration, and the temperature required to reach the optimization conditions to remove CV dye from the aqueous solution. Different kinetics and isotherm models were applied to the experimental data to explain the mechanism of the adsorption process. The adsorption of CV on Ag-Cell-NCMs follows the pseudo-second order, and the best-fit isotherm was the Langmuir isotherm. The new composite was tested for the possibility of dye desorption and ability to be reused several times, and we found that the new nanocomposite can be reused for multiple adsorptions and there is a possibility of dye desorption.

## 1. Introduction

Wastewater contaminated with dyes is a growing problem in many industries, particularly in the textile industry. This industry uses large amounts of water to rinse and dye fabrics, resulting in a significant amount of wastewater discharge. These effluents cause severe environmental contamination, leading to harmful effects on both humans and aquatic ecosystems. The chemicals in the dye waste can cause a range of issues, from changing water color and reducing transparency to harming aquatic life and degrading water quality. Over the world, many factories such as printing, food, leather, paper, and textile produce more than 100, 0000 tons of azo dyes, dyes, and dyestuff that discharge into the environment [1]. About 70% of the dyes present in the wastewater are azo-dyes, which contain the bonds (R1–N-N–R2) and their derivatives, which are toxic and mutagenic to microorganisms [2]. This has led to extensive research on finding effective and eco-friendly ways to treat wastewater contaminated with dyes. The physicochemical methods are efficient and fast but do not generally work due to high costs and the form of the complex sludge [3]. So, we should be searching for a new method to remove dye from wastewater, and nanocellulose has been exhausted as an adsorbent for many wastewater treatments, such as dyes and heavy metals [4]. Peanut husk is an important agro-waste, which is produced in high amounts every year [5]. Of the total dry weight, peanut husk contains about 35.7% cellulose and 18.7% hemicellulose, so it can be considered a potential applicant for cellulose extraction [6]. Cellulose nanocrystals are extracted from peanut husks by two methods, including alkali hydrolysis and sulfuric acid hydrolysis, and bleaching by sodium chlorite or hydrogen peroxide [7]. The synthesis of nanocellulose and cellulose nanocomposites can be a promising approach to developing efficient and cost-effective wastewater treatment strategies. Nanocomposites have a variety of benefits and advanced materials because of their unique characteristics. Several advantages of nanocomposites can be attributed to their special qualities [8]. The idea of nanocomposites involves using many resources in which at least one material is in the nanometer range [9]. High-performance materials with unique features include nanocomposites [8]. The combining of inorganic nanoparticles (Ag, Cu, Au, Cd, etc.) only or with polymers by supporting their homogeneous combination to form nanocomposite materials has been broadly utilized [10].

*Azadirachta indica* leaf extract can be used to create Ag-NPs, which is an inexpensive, quick, and reagent-free method of creating phytoremediation products [11] Silver nitrate solution was utilized to create Ag-NPs using *Azadirachta indica* leaf extract. The strategy turned out to be remarkably simple, affordable, and successful [12]. *Azadirachta indica* plant extract was used to synthesize Ag-NPs, which exhibited high chromium (Cr) removal [13].

Peanut husk is a type of agricultural waste that exhibits remarkable adsorption abilities for anionic dye from aqueous solutions [14]. Peanut shells treated with microwave irradiation and pyrolysis produce an adsorptive material with intriguing properties [15]. Microcrystalline cellulose (MCC) made from groundnut shells is regarded as an efficient adsorbent for removing environmentally harmful dyes [16].

Potential solutions resulting in effective and environmentally friendly materials for the monitoring and treatment of water contamination include Ag-NP–cellulose hybrid composites. Ag-NP–cellulose hybrids have the dual benefits of being simple to make from recycled materials, inexpensive, and potentially reusable, as well as being eco-safe when built appropriately [17]. Ag/Fe_3_O_4_/CNC is an effective catalyst for eliminating organic harmful contaminants from polluted water [18].

This work is intended to highlight the achievement of the green synthesis of Ag-NPs using *Azadirachta indica* leaf extract. We use an agro-waste, peanut husk, as a source of cellulose. We load Ag-NPs onto cellulose to form nanocomposite materials using eco-friendly and simple methods, then evaluate the efficiency of nanocomposites for the removal of dyes from aqueous solutions. The proposed research will help to develop new technologies that are eco-friendly and cost-effective and recycle agro-waste, leading to a healthier environment for future generations.

## 2. Materials and Methods

### 2.1. Materials

The peanut husks were obtained from a local market (Jeddah, Saudi Arabia) and were used as the raw source of cellulose in this study. Fresh neem leaves (*Azadirachta Indica*) were collected from the neem trees in Jeddah and used as a reducing agent to prepare silver nanoparticles. Chemical reagents used were obtained from Sigma Aldrich Company and utilized without additional chemical refining, including sodium hydroxide (NaOH; 0.5 M, and 0.1 M), nitric acid (HNO_3_; 69%), ethanol (Et OH; 96%), sodium hypochlorite solution (Na OCl; 10%), acetic acid glacial (CH_3_COOH), silver nitrate (AgNO_3_; 1 mM), and hydrochloric acid (HCl; 0.1 M). Crystal violet dye (CV) (C.I. 42555, prod: 34024, chemical formula = C_25_H_30_ClN_3_, FW = 408 g/mol, λmax = 578 nm, nature = basic dye) was used as an adsorbate. A stock solution of CV (500 mg/L) was prepared in distilled water and was diluted to prepare different working solutions (1–50 mg/L). The chemical structure of CV and its electronic spectrum are shown in Figure S1 (Supplementary Materials).

### 2.2. Methods

#### 2.2.1. Extraction of Microcrystalline Cellulose from Peanut Husk

Microcrystalline cellulose (MCC) was extracted from peanut husk (PNH) using a method reported in the literature with some modifications [19]. First, the dust and impurities were removed from PNH by washing with distilled water several times, then it was dried for 24 h in the oven at 60 °C. The dry PNH was ground into a powder using an electronic mill and sieved into 40–60 mesh (250 μm) to produce uniform particles. About 25 g of PNH powder was treated with NaOH (750 mL, 0.5 M) overnight with continuous stirring at 90 °C. The formed sark slurry was discarded, and the residue was washed with distilled water several times and then dried. The process of alkali treatment was repeated one more time. The dried alkali-treated powder (PNH-OH) was refluxed with a 20% mixture of HNO_3_ and EtOH (*v*/*v*) for 7 h until the color of the mixture changed from brown to yellow. The yellow product was then filtered and washed with cold distilled water until the filtrate became neutral. Next, the yellow color of the extracted MCC was bleached with NaOCl and 10 drops of acetic acid. Finally, the MCC was dried in an oven at 60 °C overnight.

#### 2.2.2. Green Fabrication of Ag-NPs Using *A. indica*

Silver nanoparticles (Ag-NPs) were fabricated from 1 mM AgNO_3_ solution by using the aqueous extract of *A. indica* as a reducing agent, as described in the literature [20]. The plant aqueous extract was prepared using 10 g of the clean and dried chopped *A. indica* leaves. The *A. indica*-chopped leaves were boiled in 75 mL of distilled water for 10 min. The extract mixture was then cooled and filtered using Whatman 1 filter paper. The aqueous extract was then stored at 4 °C in a covered baker until use. To fabricate the Ag-NPs, 95 mL of 1 mM of AgNO_3_ was added dropwise to 5 mL of *A. indica* and stirred with a magnetic stirrer at 80 °C for 1 hr. The color of the solution changed gradually from light green to dark brown, which indicates the formation of Ag-NPs in the solution.

#### 2.2.3. Fabrication of the Ag-Cellulose Nanocomposite (Ag-Cell-NCMs)

An appropriate amount of extracted microcellulose (0.5, 1.0, 1.5, and 2.0 g) was mixed with the Ag-NPs that were fabricated in the previous section and stirred forcefully for 24 h. After that, the resulting particles were taken out by centrifuging at 10,000 rpm. The nanocomposite particles (Ag-Cell-NCMs) were dried in an oven at 60 °C for 24 h and characterized.

### 2.3. Characterization

#### 2.3.1. Characterization of Ag-NPs Using UV-Vis Spectroscopy

The fabrication of Ag-NPs using an aqueous extract of *A. indica* was followed at different times by measuring the UV-Vis absorption spectra of periodic aliquots of 3 mL. All UV-Vis absorption spectra were recorded using a Shimadzu UV-1800 spectrophotometer (Shimadzu, Kyoto, Japan) with a 1.0 cm quartz cell in the scan range of 200–800 nm.

#### 2.3.2. Characterization of Cellulose and Ag-Cell-NCMs

The functional groups of the raw PNH, alkali-treated PNH, extracted MCC, Ag-Cell-NCMs, and Ag-Cell-NCM-adsorbed CV dye were identified using a Frontier Fourier transform infrared (FT-IR) spectrophotometer (Perkin-Elmer, Waltham, MA, USA) with KBr disks in the range of 4000–400 cm^−1^. The elemental compositions of the Ag-Cell-NCMs before and after the adsorption of CV dye were investigated using a field emission scanning electron microscope (SEM) equipped with energy-dispersive spectroscopy (EDS) (JEOL JSM-6510/v, Tokyo, Japan). The particle size and shape of Ag-Cell-NCMs and Ag-Cell-NCM-adsorbed CV dye were investigated by transmission electron microscopy (TEM), (JEOL JSM-6510/v, Tokyo, Japan). Zeta potential analysis afforded details of the stabilization of Ag-Cell-NCMs and Ag-Cell-NCM-adsorbed CV dye, measured using Malvern Zeta size Nano-Zs90 (Malvern, Westborough, PA, USA). The crystallinity of Ag-Cell-NCMs and Ag-Cell-NCM-adsorbed CV dye was estimated using an X-ray diffractometer (PAN Analytical X-Pert PRO, spectral plc, Almelo, The Netherlands).

### 2.4. Batch Adsorption Experiment

The efficiency of Ag-Cell-NCMs for the removal of dyes from aqueous solutions was studied by a batch adsorption experiment method. A known concentration of CV dye solution (10 mL) was transferred in a 50 mL Erlenmeyer flask and then mixed with a known amount of Ag-Cell-NCMs. The pH of the mixture was adjusted to the desired level, using 0.1 M HCl and 0.1 M NaOH. The mixture was shaken at a constant speed using a shaker (Stuart SSL1, UK) for the required time at room temperature (25 ± 2 °C). After reaching the equilibrium, the solution was separated from the adsorbent by centrifuging at 4000 rpm for 10 min, and the separated solution was analyzed for CV concentration using a UV-Vis spectrophotometer. A reagent blank was treated in the same manner. Several crucial parameters were studied by sequentially modulating, including nanocomposite ratio (0.5, 1.0, 1.5, and 2.0 g extracted MCC to Ag-NPs), nanocomposite dose (1.0–100 g/L), initial CV dye concentration (1.0–25 mg/L), contact time (10–180 min), and pH (2.0–10.0).

The efficiency of the CV dye removal was calculated using the following equation:

(1) $$\%\;Removal=\frac{C_{0}-C_{e}}{C_{0}}\times 100$$

where C_0_ is the initial concentration of the CV (mg/L), and C_e_ is the concentration of CV (mg/L) in the solution at the sorbate-sorbent equilibrium stage. The adsorption capacity at the equilibrium stage (q_e_) is calculated by the equation:

(2) $$q_{e}=\frac{V(C_{0}-C_{e})}{w}$$

where q_e_ is the adsorption capacity of the adsorbent (mg/g), V is the volume of the reaction mixture (L), w is the weight of the Ag-Cell-NCMs used (g), and C_0_ and C_e_ are the initial and equilibrium concentrations of adsorbate (CV dye), respectively (mg/L). Experiments were performed in triplicate to ensure the reproducibility of the process, and further, the average values were applied for the calculation of experimental data.

### 2.5. Desorption Study

A desorption process was studied to determine the possibility of recycling/regenerating the composite, which is important for practical applications. For this, the Ag-Cell-NCM-adsorbed CV dye was dried at 60 °C and then mixed with 0.2, 0.4, 0.6, 0.8, or 1.0 mol/L NaCl solutions for 24 h with stirring. Each mixture was centrifuged (4000 rpm) to separate the adsorbent, and the dye desorbed was estimated spectrophotometrically. The percentage of CV dye desorbed into NaCl was calculated using the following equation:

(3) $$Desorption\%=\frac{R}{D}\times 100$$

where R and D are dye-recovered (desorbed) and dye-adsorbed, respectively.

## 3. Results and Discussion

### 3.1. Characterization of Green Fabricated Ag-NPs

**Figure 1:**
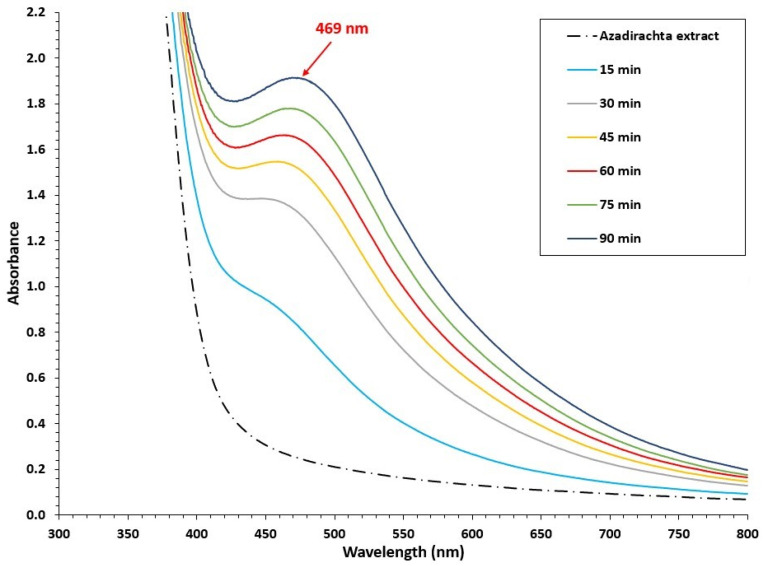
The UV-Vis absorption spectra of Ag-NPs at different times after being fabricated by *A. indica*.

Figure 1 displays the UV-Vis absorption spectrum of the aqueous extract of *A. indica* and the absorption spectra of Ag-NPs green-fabricated by *A. indica* leaves at different times. It appears that *A. indica* extract has no band in the visible region. Meanwhile, the fabrication of the Ag-NPs was confirmed by the appearance of a new band in the visible region as well as a change in the color of the solution (from light green to brown). The λmax of the nanoparticles band is 469 nm after 90 min of fabrication (Figure 1). Moreover, the measured absorbance of the Ag-NPs after fabrication increased with increasing time (from 15 to 90 min), as shown in Figure 1, which is attributed to the increase in the deep brown color as a result of the formation of more Ag-NPs [21]. The UV–Vis spectra of biosynthesized Ag-NPs at different reaction times exposed characteristic absorption bands in the range of 400–500 nm due to the surface plasmon resonance (SPR) of Ag-NPs [22]. The absorption peak of the ultraviolet-visible spectrum of the silver nanoparticles fabricated by *Azadirachta indica* was observed at 445 nm [23].

### 3.2. Characterization of Cellulose, and Ag-Cell-NCMs

#### 3.2.1. FTIR Spectroscopy

Table 1 and Figure S2 demonstrate the FTIR absorption peaks of PNH, PNH-OH, MCC, Ag-Cell-NCMs, and Ag-Cell-NCM-adsorbed CV dye. The results show that there are 16 peaks were noticed in the PNH spectrum, 14 peaks in the PNH-OH spectrum, 11 peaks in the MCC spectrum, and 10 peaks in both spectra of the Ag-Cell-NCMs and the Ag-Cell-NCM-adsorbed CV dye. There are fewer modifications in peak positions (wavenumbers, cm^−1^), which denotes a lesser change in the chemical structures of Ag-Cell-NCMs after and before dye adsorption. There was no difference in cellulose composition after and before dye adsorption, as proved by FT-IR analysis [24]. It was reported that the absorbance pattern demonstrated by the FT-IR of cellulosic olive stone biomass did not change significantly after the adsorption of MB [25].

#### 3.2.2. Energy Dispersive X-ray Measurements

**Figure 2:**
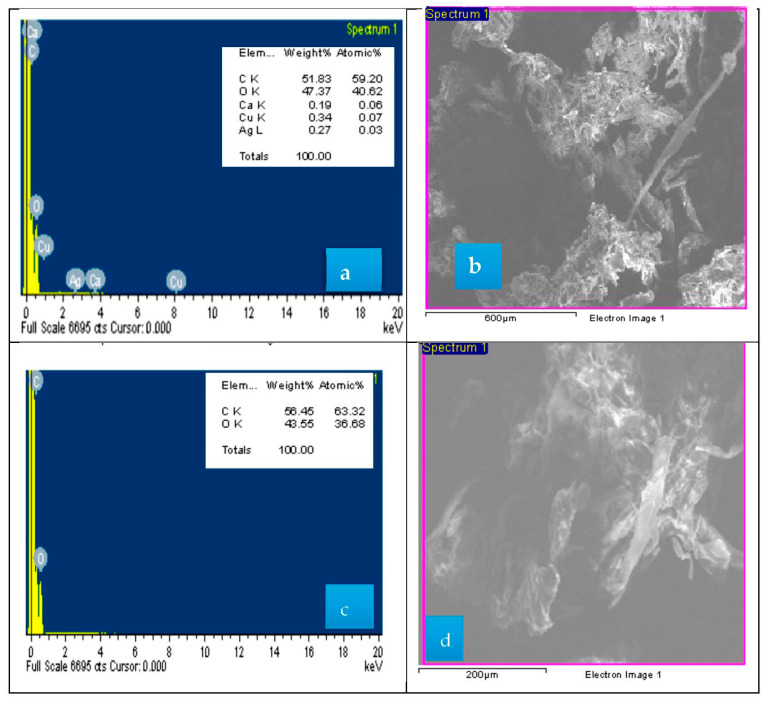
EDX analysis of, Ag-Cell-NCMs (**a**), and Ag-Cell-NCM-adsorbed CV dye (**c**), and SEM images of Ag-Cell-NCMs (**b**), and Ag-Cell-NCM-adsorbed CV dye (**d**).

The energy dispersive X-ray (EDS) measurements and SEM images of the Ag-Cell-NCMs and Ag-Cell-NCM-adsorbed CV are shown in Figure 2. The SEM image of the nanocellulose denotes that the nanocellulose is a nanofiber with a smooth surface and has a uniform appearance, in agreement with Alsaiari et al. [42]. The EDS and SEM results of the Ag-Cell-NCMs are shown in Figure 2a,b, respectively. Carbon, oxygen, calcium, copper, and silver are present in the nanocomposite. The carbon and oxygen are predominant at 51.83 and 47.37 weight%, respectively. On the other hand, the silver is present in low weight (0.27%), which may be due to the low amount of nano-silver used in the fabrication of the nanocomposite compared to that of cellulose (17 mg Ag-NPs fabricated by *A. indica* to 2.0 g of MCC). As reported in the literature, the greatest components of Ag-cellulose nanocomposites derived from green alga *Ulva lactuca* were carbon and oxygen [43]. The EDS analysis for the Ag-cellulose nanocomposite used for the adsorption of CV dye is shown in Figure 2c, where it appeared that the silver content disappeared, which may be due to the adsorption of CV dye on the composite surface. The same results were obtained for the Ag-cellulose nanocomposite used for the adsorption of malachite green dye, where the composition of silver reduced from 1.49% to 0.15% after the adsorption of malachite green [44]. The Ag-Cell-NCM-adsorbed CV dye is aggregated and complicated, as shown by the SEM image in Figure 2d. Gatenholm and Khemm mentioned that oxygen and carbon are the dominant compositions of bacterial and plant nanocellulose [45]. Also, the basic compositions of conocarpus fiber nanocellulose were oxygen and carbon [46].

#### 3.2.3. TEM Image

**Figure 3:**
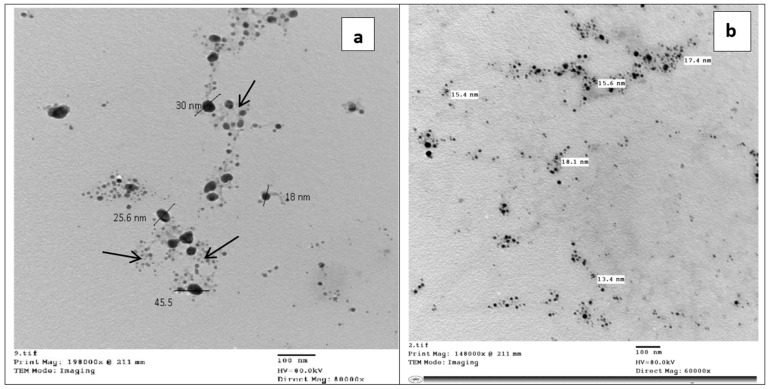
TEM images of Ag-Cell-NCMs (**a**), and Ag-Cell-NCM-adsorbed CV dye (**b**).

Figure 3a,b shows the TEM images of Ag-Cell-NCMs and Ag-Cell-NCM-adsorbed CV dye, respectively. For Ag-Cell-NCMs, the TEM image displays that the nanoparticles are spherical, and their size ranges from 18 to 45 nm, which may be related to silver nanoparticles, and arrows refer to embedded nanocellulose (Figure 3a). The size of Ag-Cell-NCMs particles was reduced after the adsorption of CV dye and ranges from 13.4 to 17.4 nm, and there a faint color may be due to the nanocellulose. The Ag-NPs were randomly distributed in nanocellulose, and CV dye was adsorbed on Ag-NPs. The same results have been reported for hydrothermally synthesized Ag-nanocellulose, where the TEM observation indicated a spherical and random distribution on nanocellulose fibrils [47]. Silver nanoparticles loaded in nanocellulose had a spherical shape and were dispersed on the surface of cellulose nanofiber uniformly, and the average size was 25 nm [48].

#### 3.2.4. Zeta Potential Analysis

**Figure 4:**
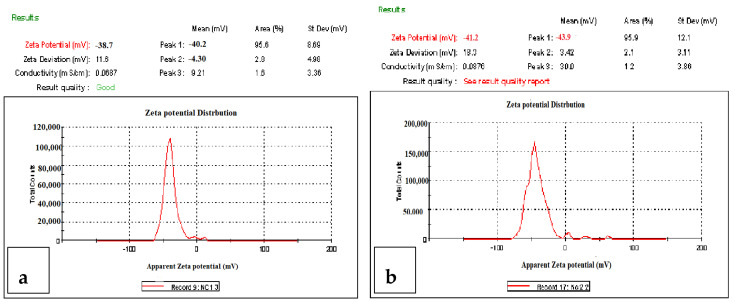
Zeta potential of Ag-Cell-NCMs (**a**), and Ag-Cell-NCM-adsorbed CV dye (**b**).

A Zeta potential analysis of Ag-Cell-NCMs and Ag-Cell-NCM-adsorbed CV dye is demonstrated in Figure 4a,b, respectively. It is observed from the figure that the Zeta potential analysis of Ag-Cell-NCMs has a negative charge (−38.7 mV). Also, Ag-Cell-NCM-adsorbed CV dye has a negative charge (−41.2 mV). These results denote that both Ag-Cell-NCMs and its absorbed CV dye have good stability, and the nanocomposite stability was increased after the adsorption of CV dye. According to Kumar et al., if Zeta potential values range between ±30 and ±40 mV, this indicates the medium stability of the composite, and if they range between ±40 and ±60 mV, the composite has good stability [49].

#### 3.2.5. XRD Patterns

**Figure 5:**
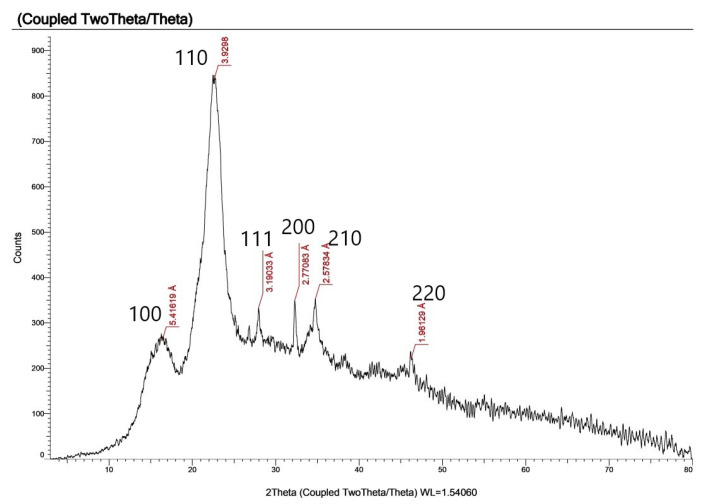
XRD patterns of Ag-Cell-NCMs.

The crystalline structure of Ag-Cell-NCMs and Ag-Cell-NCM-adsorbed CV dye was investigated by XRD measurements (Figure 5 and Figure S3). The two cellulose nanocomposite samples before and after the adsorption of CV dye displayed the same diffraction peaks at nearly the same 2θ angles. The peaks that appeared at 2θ were 16.353, 22.608, 27.944, 32.282, 34.766, and 46.252°, which could be attributed to the lattice planes 100, 110, 111, 200, 210, and 220, respectively. The sharp bands over 2θ may be attributed to the cellulose nanocomposite being crystalline. No significant changes in the crystalline peaks before and after dye absorption were reported in the research [50].

### 3.3. pH Zero-Point Charge (pHzpc)

The adsorption ability of the adsorbent at different pH levels and the distribution of the charges on the surface could be evaluated by determining the pH zero-point charge (pHzpc) [51]. The pHzpc of the Ag-Cell-NCMs was determined utilizing a solid addition method [52]. Figure S4 shows the obtained results of pHzpc determination, where the pHzpc of the Ag-Cell-NCMs was found to be 4.65. Consequently, when the pH value of the adsorption system is more than the pHzpc value, the absorbent surface becomes negatively charged and the adsorption of cations is favored. Conversely, when the pH value is lower than pHzpc, the adsorbent surface becomes positively charged as a result of the protonation of functional groups, which leads to an electrostatic attraction between the anionic and adsorbent [53]. Thus, the adsorption of cationic dyes such as CV dye is favored to occur on Ag-Cell-NCMs at a pH more than the pHzpc.

### 3.4. Adsorption Study

#### 3.4.1. Screening of Adsorbents

**Figure 6:**
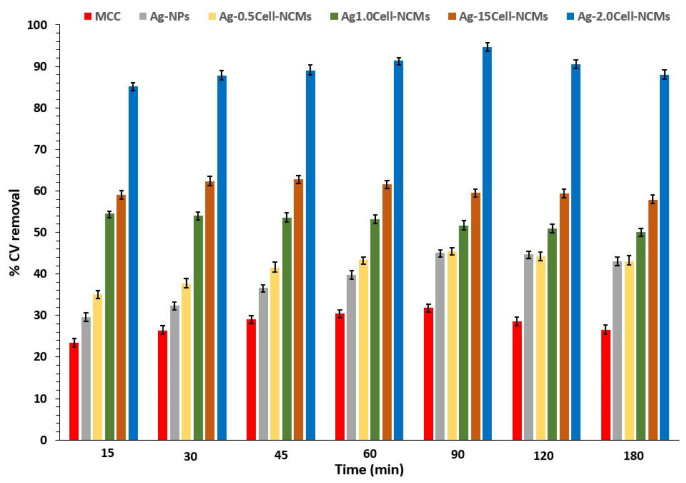
Screening of MCC, Ag-NPs, and fabricated Ag-Cell-NCMs for CV dye removal (Conditions; 0.02 g adsorbent doses, pH 6, temperature 25 °C, dye initial concentration 15 mg/L, contact time 15–180 min and shaking speed 200 rpm).

Different compositions of silver microcellulose nanocomposites were synthesized using different amounts of extracted microcellulose with the same amount of fabricated Ag-NPs solution. The extracted MCC, isolated Ag-NPs, and fabricated Ag-Cell-NCMs were screened for CV removal under experimental conditions of 0.02 g adsorbent, pH 6, 25 °C temperature, 15 mg/L CV dye initial concentration, and 200 rpm shaking speed for different periods, and the obtained results are illustrated in Figure 6. The maximum removal percentages obtained by extracted MCC, isolated Ag-NPs, Ag-0.5Cell-NCMs, Ag-1.0Cell-NCMs, Ag-1.5Cell-NCMs, and Ag-2.0Cell-NCMs were 31.9 ± 0.75, 45.11 ± 0.69, 45.5 ± 0.77, 54.5 ± 0.55, 62.8 ± 0.89, and 94.6 ± 1.10%, after 90, 90, 90, 15, 45, and 90 min, respectively. Among the investigated adsorbents, the Ag-2.0Cell-NCM prepared by mixing 2.00 g MCC and 10.79 mg Ag-NPs showed the highest removal efficiency compared with other Ag-Cell-NCM compositions. On the other hand, the results indicate that the removal of the CV dye is higher when using fabricated nanocomposites rather than extracted cellulose or Ag-NPs alone. It is also noted from Figure 6 that the CV removal using Ag-NPs and the Ag-0.5Cell-NCMs after 90 min were close. However, the increase in the cellulose ratio in the nanocomposite increases the adsorption of the dye, which may be due to the increase in the functional groups on the surface of the adsorbent. Previous studies disclose that nanocomposites better adsorb pollutants from wastewater in comparison with native agricultural adsorbents since nanocomposites provide a compatible matrix that gives rise to the adsorption properties [54]. The Ag-2.0Cell nanocomposite was chosen for the adsorption studies and is abbreviated as Ag-Cell-NCMs in the next sections.

#### 3.4.2. Effect of pH

**Figure 7:**
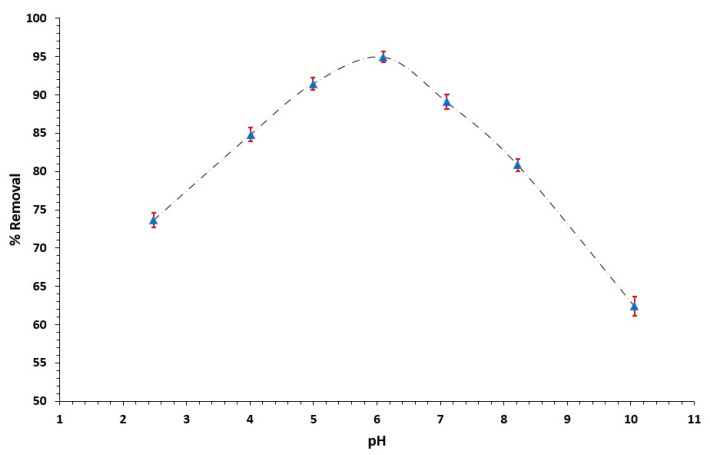
Effect of pH on CV dye removal by Ag-Cell-NCMs (Conditions; 0.02 g adsorbent doses, pH 2–10, temperature 25 °C, dye initial concentration 15 mg/L, contact time 90 min, and shaking speed 200 rpm).

The removal of CV dye using Ag-Cell-NCMs as an adsorbent was investigated at different pH levels (from 2 to 10). The obtained results are presented in Figure 7, where the removal percentages were increased from pH 2 to 6 and then decreased from pH 7 to 10. The results demonstrate that the adsorption process decreased at low and high pH levels, while the maximum CV removal was recorded at a moderate pH level. It is well known that both the degree of dye ionization and the charge of the adsorbent surface are dependent on the pH of the solution [55]. Moreover, the pHzpc value of the composite was found to be 4.65, so the surface of the composite has a positive charge below this pH, which leads to an electrostatic repulsion between the adsorbent surface and the positive ions of the dye. Also, the low adsorption at low pH could be attributed to the competition between the hydrogen ions and the CV ions for binding sites [56]. In contrast, as the pH increases, the composite surface sites become more negatively charged, thereby resulting in the increased adsorption of CV dye due to increasing electrostatic attraction between the negatively charged surface and the positive ions of the dye [53]. At a higher pH, the removal of CV dye decreased, which could be attributed to the decrease in the solubility of the dye. A similar trend was reported in the literature: that at low and high pH levels, the adsorption of the dye decreased, while the maximum adsorption was achieved at a pH of around 6 to 8 [56].

#### 3.4.3. Effect of Ag-Cell-NCMs Dose

**Figure 8:**
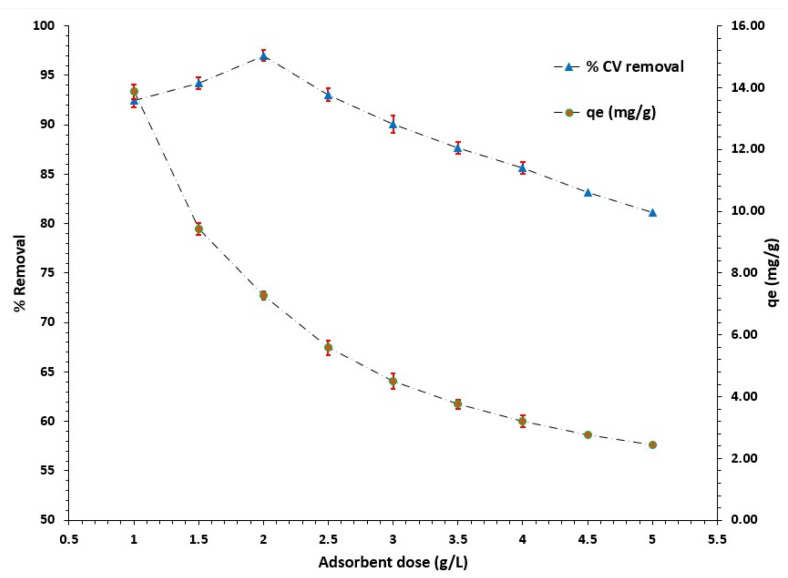
Effect of adsorbent dose on CV dye removal by Ag-Cell-NCMs (Conditions; 0.01–0.05 g adsorbent doses, pH 6, temperature 25 °C, dye initial concentration 15 mg/L, contact time 90 min and shaking speed 200 rpm).

The % removal of CV dye and the adsorption capacity (qe) were determined using different doses of Ag-Cell-NCMs in the range of 1.0–5.0 g/L, and the results are shown in Figure 8. The increase in the adsorbent doses significantly affects the removal of CV dye, where the removal of the CV dye was increased when increasing the adsorbent dose, reaching the maximum removal at 2.0 g/L. Then, with greater Ag-Cell-NCMs doses, the removal of the dye was decreased. One possible explanation for this decrease in adsorption capacity with dose is the overlapping of active moieties, which reduces the surface area available for binding [57].

#### 3.4.4. Effect of Contact Time

**Figure 9:**
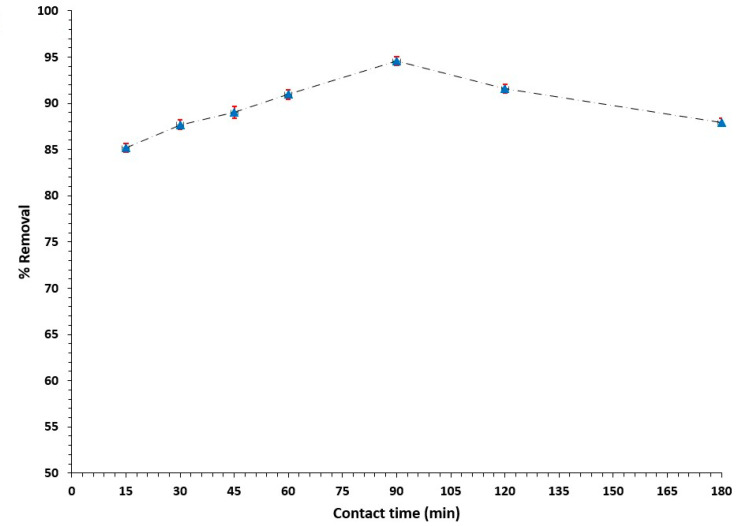
Effect of contact time on CV dye removal by Ag-Cell-NCMs (Conditions; 0.02 g adsorbent dose, pH 6, temperature 25 °C, dye initial concentration 15 mg/L, contact time 15–180 min, and shaking speed 200 rpm).

Figure 9 shows the effect of contact time on the removal of CV dye using Ag-Cell-NCMs. The % removal at the beginning of the process increased with increasing contact time, which can be attributed to the available large surface area of Ag-Cell-NCMs and the presence of more free binding sites. The maximum removal was achieved after 90 min (94.6 ± 0.43%), as shown in Figure 9. The binding sites became saturated beyond this time with adsorbed CV ions. These already-occupied binding sites repel the upcoming CV ions, and the removal of CV dye then decreases slightly with increasing time [58]. Moreover, the adsorption process of CV dye using Ag-Cell-NCMs was studied for 24 h, and the % removal was about 97%, which indicates the efficiency of the removal, and the time has no effect after reaching the equilibrium. The same behavior was reported after reaching the maximum adsorption, and over time, the adsorption process was decreased [59].

#### 3.4.5. Effect of Initial Dye Concentration

**Figure 10:**
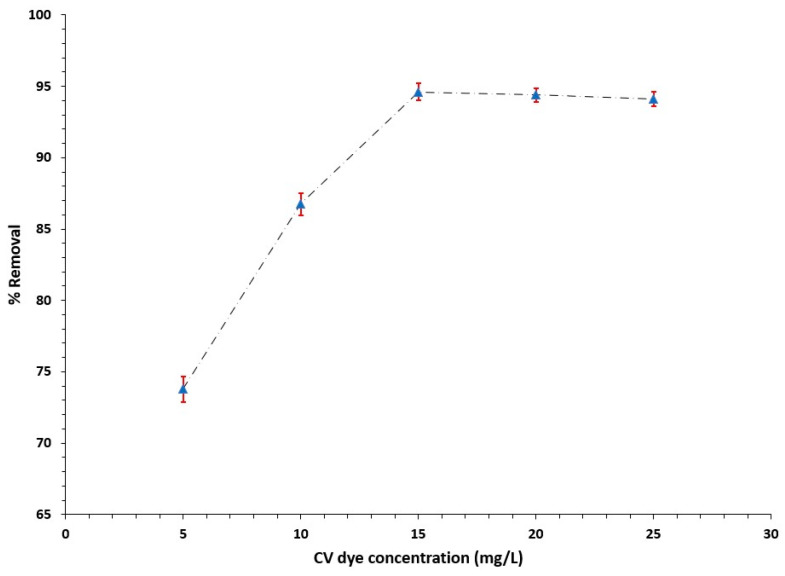
Effect of initial dye concentration on CV dye removal by Ag-Cell-NCMs (Conditions; 0.02 g adsorbent dose, pH 6, temperature 25 °C, dye initial concentration 5–25 mg/L, contact time 90 min, and shaking speed 200 rpm).

The effect of initial dye concentration in the 5.0–25 mg/L range was investigated, and the results are shown in Figure 10. As noticed in Figure 10, the removal of the CV dye was increased by increasing the initial concentration from 5.0 to 15 mg/L in an almost linear behavior, which could be due to the greater interaction between the CV molecules and the active sites on the surface of the composite. At higher initial concentrations, the % removal became almost constant with little decrease due to the saturation of the active sites on the composite surface [59]. The same behavior was reported for dye adsorption using different composites [60].

#### 3.4.6. Effect of Temperature

**Figure 11:**
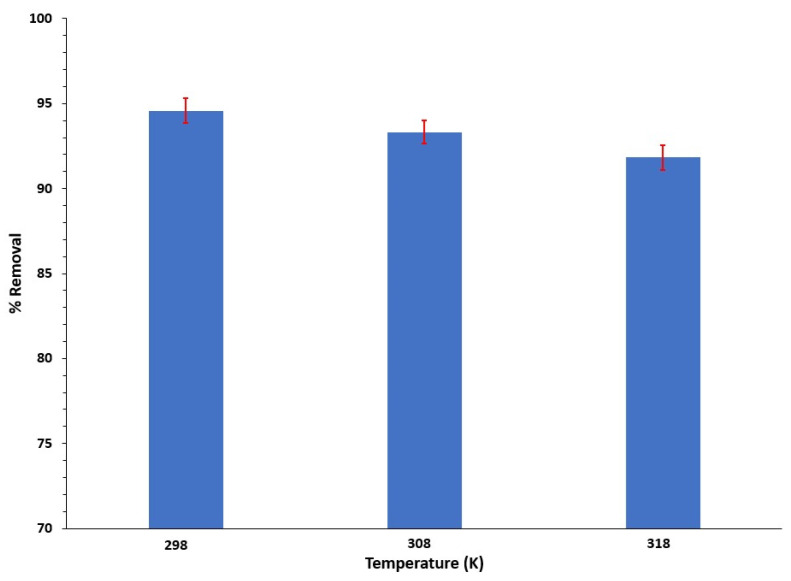
Effect of temperature on CV dye removal by Ag-Cell-NCMs (Conditions; 0.02 g adsorbent dose, pH 6, temperature 298–318 K, dye initial concentration 15 mg/L, contact time 90 min, and shaking speed 200 rpm).

The change in the temperature of a system affects the isothermal capacity of the adsorbent for a specific dye. Therefore, the effect of temperature on the removal of CV dye using Ag-Cell-NCMs was determined. Figure 11 shows the % removal at three temperatures (298, 308, and 318 K). A decrease in the % removal of the dye was detected when the temperature was increased from 298 to 318 K, which indicates that the adsorption of CV dye on the composite is an exothermic process. The increase in the removal of CV dye on Ag-Cell-NCMs with increasing temperature can be attributed to the breakdown of the adsorption forces that bound the CV dye to the nanocomposite surface [53,54]. Foroutan et al. explained the decrease in the removal of CV dye with increasing temperature on an activated carbon/Fe_2_O_3_ nanocomposite based on the increase in the solubility of CV dye in an aqueous solution, which makes the interactions between the dye molecule and the solvent stronger than the adsorbent [54].

### 3.5. Kinetic Study

In this study, the experimental data from the adsorption of CV dye on Ag-Cell-NCMs were analyzed by pseudo-first-order [61], pseudo-second-order [62], and Elovich models [63] to determine the mechanism and rate of the adsorption process [62] by applying the simple linear Equations (4)–(6), respectively.

(4) $$ln\left(q_{m}-q_{t}\right)=k_{1}t+{lnq}_{m}$$

(5) $$\frac{t}{q_{t}}=\frac{1}{q_{m}}t+\frac{1}{k_{2}q_{m}^{2}}$$

where q_m_ and q_t_ (mg/g) represent the amount of dye adsorbed at equilibrium and any t time, respectively, k_1_ and k_2_ are the rate constant of pseudo-first-order and pseudo-second-order adsorption, respectively.

(6) $$q_{t}=\alpha +\beta lnt$$

where α is the initial adsorption rate (mg/g min), and β is the constant rate related to the extent of the surface coverage and activation energy for the adsorption (g/mg). The kinetic parameters of the adsorption kinetic models were calculated to evaluate the applicable model, and the results are summarized in Table 2 and Table S1. The correlation coefficient (R2), which is a numerical measure that statistically indicates the relationship between two variables, was also calculated.

**Scheme 1:**
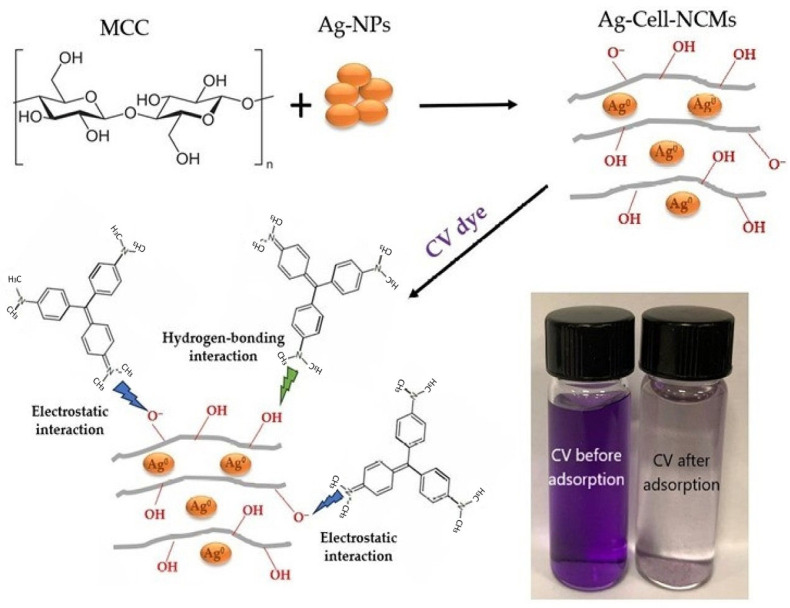
Schematic drawing for the possible interactions between the Ag-Cell-NCMs and CV dye.

As shown in Table 2 and Table S1, the correlation coefficient (R2) for the pseudo-second order has the highest value (0.999), and the calculated equilibrium adsorption capacity (qm, calc) was much closer to the experimental (qm, exp), which indicates that the adsorption of CV dye on Ag-Cell-NCMs follows the pseudo-second-order mechanism and that the rate of the adsorption process is controlled by the chemisorption process, Also, the pHzpc value for Ag-Cell-NCMs is 4.65, indicating that the surface of the composite is negatively charged at pH > 4.65. Therefore, the adsorption mechanism of the cationic dye (CV) on Ag-Cell-NCMs includes electrostatic and hydrogen bonding interactions between CV and the functional groups (−OH) on the surface of the composite, as shown in Scheme 1.

In order to demonstrate the diffusion behavior, including external and internal diffusion, we employed the intraparticle diffusion model equation from Weber and Morris [64] as follows:

(7) $$q_{t}=k_{id}t^{½}+C_{id}$$

where k_id_ is the intraparticle diffusion rate constant (mg/g min½), and C is related to the thickness of the boundary layer. The plot of qt versus t½ is shown in Figure S5. The kid and Cid are the slope and intercept of the straight-line portion of the plot, respectively. The values of kid and Cid for the interparticle diffusion model were collected in Table S1. The plot in Figure S5 has a double nature, i.e., the initial and final linear portions. The initial linear portion is related to the boundary layer diffusion effects, while the final linear portion is due to interparticle diffusion. The results indicate that the intraparticle diffusion kinetic is not the only rate-limiting step because the curve does not pass through the origin [65], and the value of the intercept of the initial linear plot, Cid, expresses the extent of boundary layer thickness [66].

### 3.6. Adsorption Isotherm

The adsorption isotherms are used to understand how adsorbate molecules are distributed between the liquid and solid phases at equilibrium, to find a suitable model that can be used for design purposes [67]. Different isotherm models, namely Langmuir, Freundlich, and Temkin, were utilized to evaluate the adsorption process of CV dye on Ag-Cell-NCMs [68]. The best-fit model was chosen based on the values of linear regression correlation coefficients, R2.

The linear form of the Langmuir isotherm equation is given by:

(8) $$\frac{C_{e}}{q_{e}}=\frac{K_{L}}{q_{m}}+\frac{C_{e}}{q_{m}}$$

where q_m_ is the maximum adsorption capacity (mg/g) corresponding to complete monolayer coverage, K_L_ is the Langmuir constant and corresponding to the energy of adsorption (L/mg), C_e_ is the concentration of CV dye at equilibrium (mg/L), and q_e_ is the adsorption capacity at equilibrium (mg/g). The q_m_ and K_L_ were calculated from the slope and intercept of the straight line obtained from plotting C_e_/q_e_ against C_e_, and the resulting values along with R2 are listed in Table 3. The dimensionless separation parameter (R_L_) is an essential term that is used to measure the favorability of the Langmuir isotherm [69], and it is defined by the following equation:

(9) $$R_{L}=\frac{1}{{1+K}_{L}C_{0}}$$

where K_L_ is the Langmuir constant and C_0_ is the initial dye concentration (mg/L). The value of R_L_ indicates the type of the isotherm to be either linear adsorption if R_L_ = 1, favorable adsorption if 0 < R_L_ < 1, unfavorable adsorption if R_L_ > 1, and irreversible if R_L_ = 0 [58]. The calculated value of R_L_ was 0.0091 (Table 3), indicating that the adsorption of CV on the composite is a favorable process.

The linearized of Freundlich isotherm is given by Equation (10) [68]:

(10) $$lnq_{e}=lnK_{F}+\frac{1}{n_{F}}lnC_{e}$$

where q_e_ is the adsorption capacity at equilibrium (mg/g), C_e_ is the concentration of CV dye at equilibrium (mg/L), K_F_ is the Freundlich isotherm constant (mg/g), and 1/n is the heterogeneity intensity factor. The values of K_F_ and 1/n are determined from the intercept and slope of the plot between lnq_e_ and lnC_e_, and the results are listed in Table S2.

The linear form of Temkin isotherm is given by Equation (11) [69]:

(11) $$q_{e}=BlnK_{T}+BlnC_{e}$$

where B is the adsorption heat and K_T_ is the Tamkin constant. The constants B and K_T_ can be derived from the intercept and slope of the plot of q_e_ versus C_e_, and the values are listed in Table S2 along with R2.

Compared to the values of R2 listed in Table 3 and Table S2, the highest value of R2 was from the Langmuir isotherm model, which was approximately equal to 1 (0.9988), indicating that the Langmuir isotherm fits the adsorption of CV dye on Ag-Cell-NCMs. This assumes that the monolayer adsorption process takes place on a homogenous adsorption surface containing a finite number of identical active sites, where no further adsorption occurs as saturation is achieved [70].

### 3.7. Thermodynamic Studies

Thermodynamic studies are useful for evaluating the spontaneity of the adsorption process and for calculating the heat change of CV dye adsorption on Ag-Cell-NCMs. Thermodynamic parameters—free energy (ΔG°), enthalpy (ΔH°), and entropy (ΔS°)—were determined experimentally at three temperatures (298, 308, and 318 K), applying the following equations:

(12) $$K_{D}=\frac{q_{e}}{C_{e}}$$

(13) $$lnK_{D}=-\frac{{∆H}^{{}^{\circ}}}{RT}+\frac{{∆S}^{{}^{\circ}}}{R}$$

(14) $${∆G}^{{}^{\circ}}={∆H}^{{}^{\circ}}-T{∆S}^{{}^{\circ}}$$

where K_D_ is the equilibrium constant (L/mg), q_e_ is the amount of adsorbed dye at equilibrium (mg/g), C_e_ is the concentration of dye in the solution at equilibrium (mg/L), T is the absolute temperature (K), and R is the universal gas constant (8.314 J/K mol). By plotting the values of lnK_D_ against 1/T (Equation (13)), ΔH° and ΔS° values were derived from the slope and intercept of the plot. The results are given in Table 4, where ΔG° recorded negative values, indicating the spontaneity of the adsorption process, and the spontaneity was increased with increasing temperature. According to Hussin and Kassim, an ΔG° value that is around −20 kJ/mol or less negative confirms the physisorption mechanism, while a ΔG° value of around −40 kJ/mol or more negative confirms the chemisorption mechanism [71]. Based on this, the CV dye molecules were chemically adsorbed on the surface of Ag-Cell-NCMs since the values of ΔG° were −105–−109 kJ/mol, which agrees with the kinetic and isotherm studies. Furthermore, the ΔH° recorded a negative value, which indicates the exothermic nature of the process and confirms the chemisorption mechanism [72]. The randomness at the adsorbent–adsorbate interface during adsorption was confirmed through the positive value of ΔS° (Table 4).

### 3.8. Desorption Study

**Figure 12:**
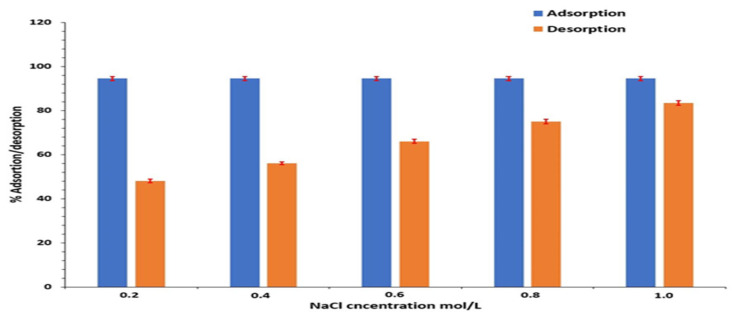
Desorption study of CV dye from the Ag-Cell-NCMs using different concentrations of NaCl solutions.

Figure 12 shows a comparison between the percentages of the adsorption and desorption process of the dye using different concentrations of NaCl solution. It was found that the maximum CV desorption is 83.40 ± 1.15%, and it was achieved by using 1.0 mol/L NaCl solution for five (adsorption/desorption) cycles, which indicates that the new nanocomposite can be reused for multiple adsorptions and suggests the possibility of dye desorption.

### 3.9. Comparative Study

The removal efficiency, desorption percentage, and time of equilibrium of previously published cellulose-based Ag-NP composites were compared with the current work to evaluate the efficacy of Ag-Cell-NCMs as adsorbents for dye removal from aqueous solutions, as indicated in Table 5. As noticed in Table 5, the Ag-Cell-NCMs exhibited a high removal efficiency toward CV dye and a high desorption percentage. Their application for real sample treatment is encouraged due to their excellent removal efficiency. Moreover, this adsorbent could be tested for other contaminants.

## 4. Conclusions

This study demonstrates that an agro-waste, peanut husk, can be used for extracting microcellulose, loading it with Ag-NPs green-fabricated by *Azadirachta indica* to form Ag- Ag-cellulose nanocomposites (Ag-Cell-NCMs). The adsorption of CV dye on Ag-Cell-NCMs was investigated under different experimental conditions. The results show that adsorption increased with increasing initial concentration and contact time, while it decreased with increasing adsorbent dose and temperature. The maximum removal of the dye was achieved at pH 6.0 and 298 K and was found to be about 95%. The adsorption kinetics of CV removal by Ag-Cell-NCMs follows the pseudo-second-order model, and chemisorption was confirmed. The Langmuir isotherm better fitted the experimental adsorption results according to the highest value of the correlation coefficient (R2). The thermodynamic parameters indicated that the adsorption of CV dye is spontaneous, exothermic, and favorable. The desorption study confirmed that the synthesized Ag-Cell nanocomposite can be reused for multiple adsorptions and suggests the possibility of dye desorption. Based on the results, the synthesized Ag-Cell-NCMs are a cost-effective adsorbent for the removal of cationic dyes from aqueous solutions.

## Figures and Tables

**Table 1:** FTIR Absorption peaks that assigned to the active groups of PNH, PNH-OH, MCC, Ag-Cell-NCMs, and Ag-Cell-NCM-adsorbed CV.

Wavenumber cm^−1^	PNH	PNH-OH	MCC	Ag-Cell-NCMs	Ag-Cell-NCM-Adsorbed CV	Active Groups	References
3403	D	−3	−1	−3	−7	Stretching vibration of –O–H	[26]
2923	D	D	D	D	−1	Stretching vibration of –C–H and CH_2_	[27]
2853	D	D	D	D	D	Aliphatic saturated C–H stretching vibration in cellulose	[28]
1740	D	ND	ND	ND	ND	Stretching vibration of C=O	[29]
1638	D	−21	−7	−3	−2	H–O–H (water adsorption)	[30]
1513	D	−4	ND	ND	ND	C–H stretching vibration of lignin	[31]
1457	D	−2	D	D	−2	CH_2_ deformation stretching in lignin	[32]
1425	D	−5	D	ND	ND	C–H asymmetric deformation in methoxyl	[33]
1378	D	+5	−2	−2	−4	CH of cellulose	[34]
1324	D	−4	ND	ND	ND	CH_2_ bending	[35]
1264	D	+2	ND	ND	ND	C–O stretching vibration	[36]
1155	D	+7	+8	+10	+10	C–O–C stretching vibrations	[37]
1104	D	+4	+10	+10	+7	C–O vibrational peaks from cellulosic alcohols	[38]
1054	D	D	+7	+3	D	C–O–C asymmetric valence vibration	[39]
1036	D	−6	ND	ND	ND	C–O stretching, aromatic C–H in-plane deformation	[40]
618	D	ND	+2	D	D	ClO_4_–1 symmetric and asymmetric stretching of the free anion	[41]

PNH: peanut husk; PNH-OH: alkali-treated peanut husk; MCC: microcellulose; Ag-Cell-NCMs: nanocomposite; Ag-Cell-NCMs-CV: nanocomposite adsorbed crystal violet dye; D: Detected; ND: Not Detected.

**Table 2:** Application of pseudo-second-order model.

Kinetic Model	At 298 K
Pseudo-second-order	
k2 (g/mg min)	0.0456
q_e_-experimental (mg/g)	7.0928
q_e_-calculated (mg/g)	7.2622
R2	0.9990

**Table 3:** Langmuir adsorption parameters.

Isotherm Model	298 K
Langmuir	
K_L_ (L/mg)	7.2731
q_m_ (mg/g)	6.0570
R_L_	0.0091
R2	0.9988

**Table 4:** Thermodynamic parameters for CV dye adsorption on the Ag-Cell-NCMs.

Temperature (K)	−ΔG° (kJ/mol)	−ΔH° (kJ/mol)	ΔS° (J/mol K)
298	105.5	49.96	183.4
308	107.4		
318	109.2		

**Table 5:** Comparative study of Ag-cellulose nanocomposite adsorbents for removal of different contaminants.

Adsorbent	Absorbate	%Removal	%Desorption	Equilibrium Time (min)	Ref.
Ag-NPs/CNF composites	Malachite green	92.0	No data available	100	[59]
Ag-NPs	Methylene blue	70.0	No data available	120	[73]
Ag-NPs incorporated into the cellulose membrane	Cartap (CA) pesticides	89.7–100	No data available	20–45	[74]
Ag-NPs incorporated into the cellulose membrane	Cypermethrin insecticide	95.1–99.8	No data available	20–35	[74]
Ag-ZnO support on nanocellulose	Methylene blue	90.0	No data available	6 h under UV light	[75]
Ag-Cell-NCMs	Crystal violet	95%	83.4 with 1.0 M NaCl	90	This work

## Data Availability

The datasets spent and/or analyzed during this study are available from the corresponding author on reasonable request.

## Supplementary Materials

The following supporting information can be downloaded at: https://www.mdpi.com/article/10.3390/polym15224394/s1, Figure S1: Chemical structure of CV dye and the electronic spectrum of 15 mg/L CV. Figure S2: FTIR spectra of PNH, PNH-OH, MCC, Ag-Cell-NCMs, and Ag-Cell-NCMs-CV. Figure S3: XRD patterns of Ag-Cell-NCM-adsorbed CV dye. Figure S4: The pHzpc of Ag-MCC nanocomposite; Figure S5: Intraparticle diffusion of CV dye on Ag-MCC nanocomposite (Conditions; 0.02 g adsorbent dose, pH 6, temperature 25 °C, dye initial concentration 15 mg/L, contact time 15–90 min, and shaking speed 200 rpm). Table S1: Application of different kinetic models. Table S2: Freundlich, and Temkin adsorption parameters.
