# Supplementary material for: Study on the Isotherms, Kinetics, and Thermodynamics of Adsorption of Crystal Violet Dye Using Ag-NPs-Loaded Cellulose Derived from Peanut-Husk Agro-Waste
Source: Polymers (Basel). 2023 Nov 13;15(22):4394. doi: 10.3390/polym15224394 (PMC10674550; doi:10.3390/polym15224394)
Supplement: Supplementary file 1 [file polymers-15-04394-s001.zip › polymers-2662823-supplementary.pdf]

Study on the Isotherms, Kinetics, and Thermodynamics of Adsorption of Crystal Violet Dye Using Ag-NPs-Loaded Cellulose Derived from Peanut-Husk Agro-Waste

Ghalia Saleem Aljeddani, Reem Mohammad Alghanmi and Ragaa A. Hamouda

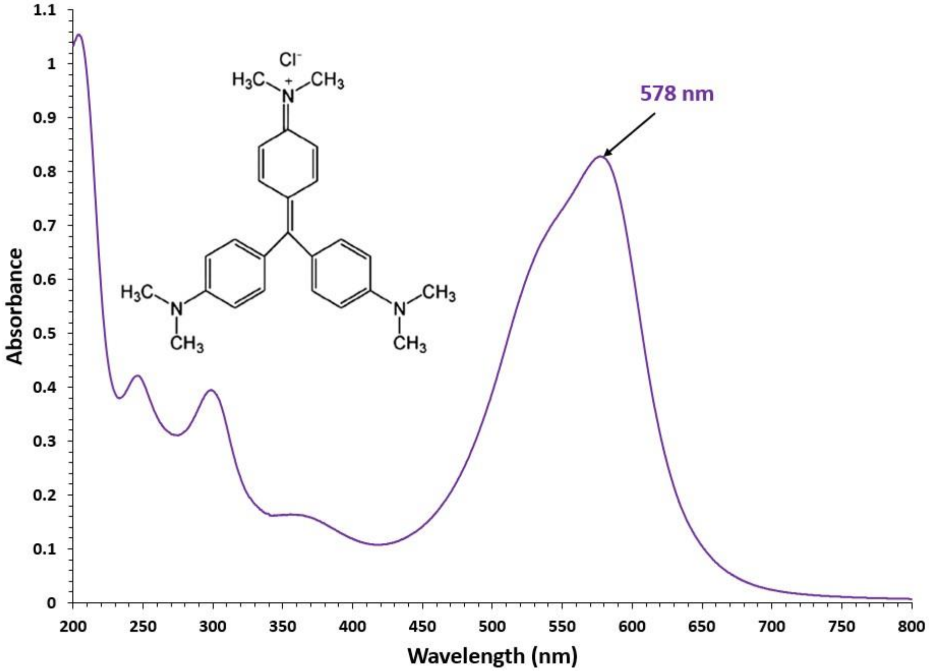

Figure S1. Chemical structure of CV dye and the electronic spectrum of 15 mg/L CV.

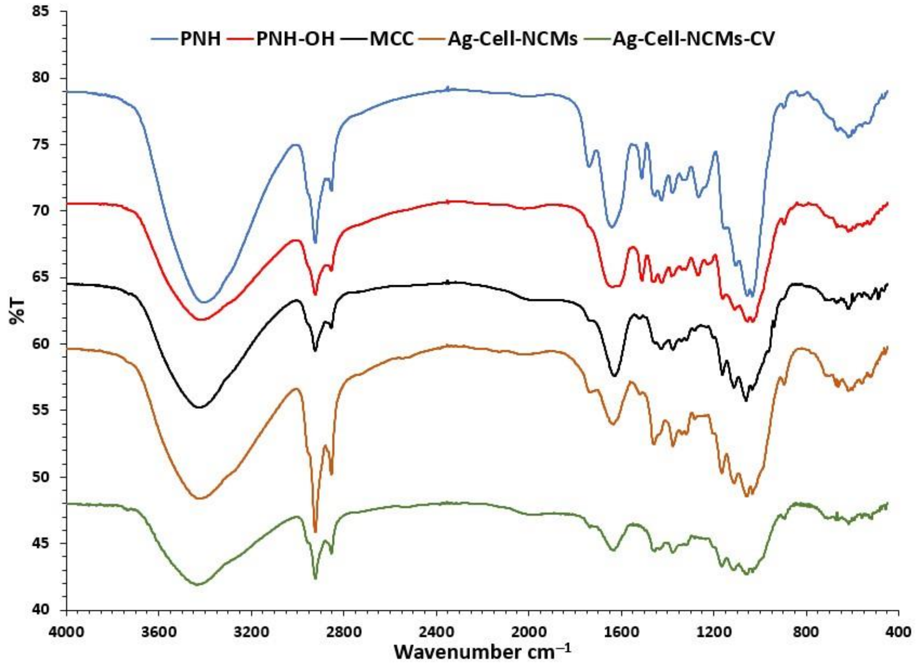

Figure S2. FTIR spectra of PNH, PNH-OH, MCC, Ag-Cell-NCMs, and Ag-Cell-NCMs-CV.

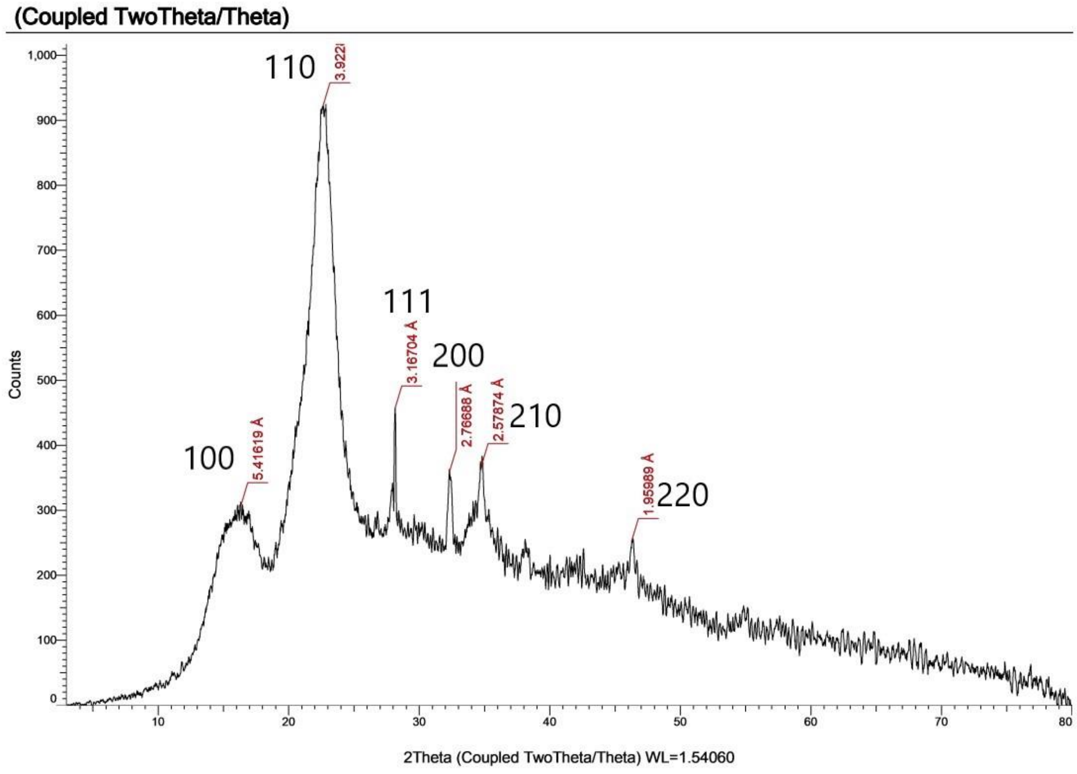

Figure S3. XRD patterns of Ag-Cell-NCMs adsorbed CV dye.

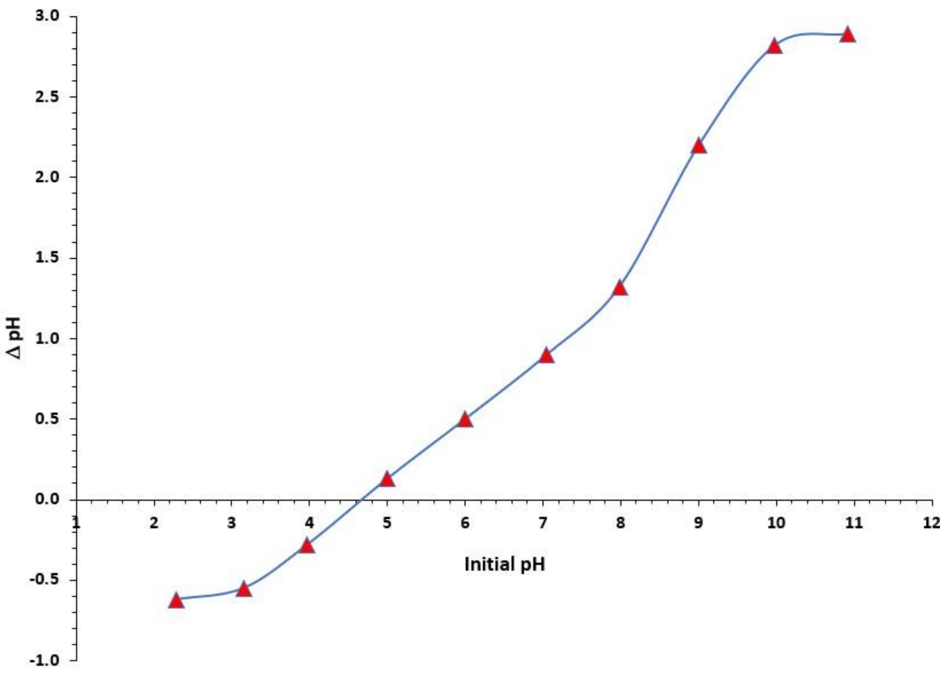

Figure S4. The pHzpc of Ag-Cell nanocomposite.

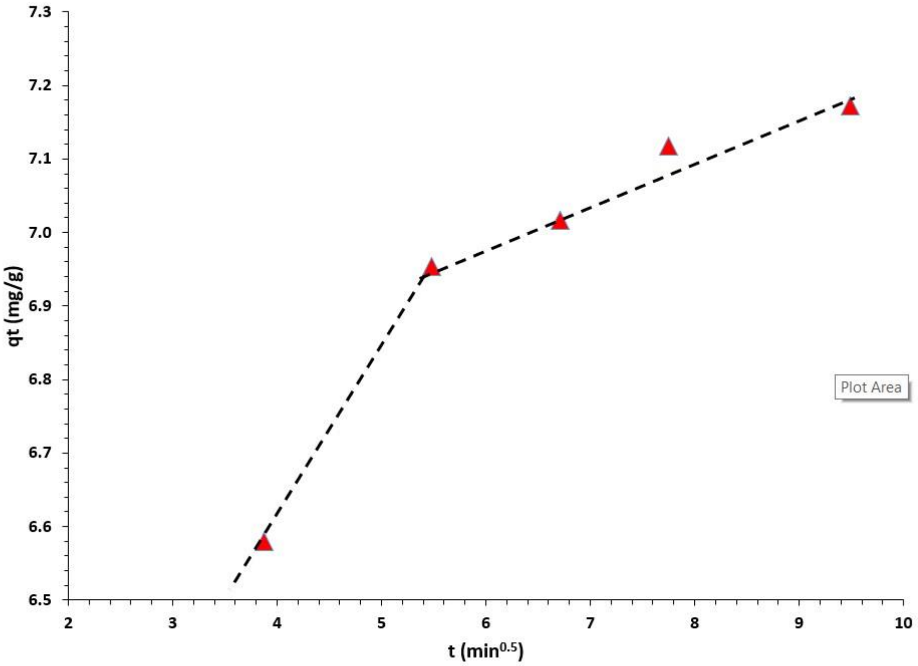

Figure S5. Intraparticle diffusion of CV dye on Ag-Cell-NCMs (Conditions; 0.02 g adsorbent dose, pH 6, temperature 25 °C, dye initial concentration 15 mg/L, contact time 15-90 min, and shaking speed 200 rpm).

Table S1. Application of different kinetic models.

Kinetic model	at 298 K
Pseudo-first-order	
k1 (1/min)	0.0205
qe-experimental (mg/g)	7.0928
qe-calculated (mg/g)	1.0281
R2	0.9823
Elovich	
β (g/mg)	0.3758
α (mg/g min)	5.3197
qe-experimental (mg/g)	7.0928
qe-calculated (mg/g)	7.0107
R2	0.9417
Intraparticle diffusion	
Kid (mg/g min½)	0.1342
Ci	6.1180
R2	0.9090

Table S2. Freundlich, and Temkin adsorption parameters.

Isotherm model	298 K
Freundlich	
Kf	12.248
1/n	0.1009
R2	0.9700
Temkin	
KT	4.89 × 10–7
B	– 0.4932
R2	0.6610
